# Supplementary material for: Skin lesion classification using ensembles of multi-resolution EfficientNets with meta data
Source: MethodsX. 2020 Mar 19;7:100864. doi: 10.1016/j.mex.2020.100864 (PMC7150512; doi:10.1016/j.mex.2020.100864)
Supplement: Supplementary file 1 [file mmc1.docx]

Dear editor,

Please find attached our method description entitled “Skin Lesion Classification Using Ensembles of Multi-Resolution EfficientNets with Meta Data” which we submit to be considered for publication in MethodsX.

We describe our method for skin lesion classification which we employed in the ISIC 2019 Skin Lesion Classification Challenge where we achieved first place in both challenge tasks (https://challenge2019.isic-archive.com/leaderboard.html). Our approach relies on multiple deep learning architectures and a combination of different techniques, including loss balancing, data augmentation strategies, and multi-resolution convolutional neural networks. The challenge’s training data is publicly available and our code is available on GitHub (https://github.com/ngessert/isic2019).

Often, the winning methods for challenges are published alongside workshop proceedings, which was not the case for the ISIC 2019 Challenge. Therefore, we would like to make our successful approach available to the scientific community through MethodsX. Generally, we believe that deep learning methods are well suited for the Journal’s aims and scope. Methods that were validated by winning public challenges are particularly interesting, as they usually introduce small but impactful design choices, method modifications, and tricks that are not very well known. Therefore, we believe that sharing our insights in MethodsX could greatly benefit the research community.

Sincerely,

Nils Gessert
